# Supplementary material for: Proteomic Analysis of the Fusarium graminearum Secretory Proteins in Wheat Apoplast Reveals a Cell-Death-Inducing M43 Peptidase
Source: J Fungi (Basel). 2025 Mar 21;11(4):240. doi: 10.3390/jof11040240 (PMC12027835; doi:10.3390/jof11040240)

**Figure S1.** Construction and identification of the *Fg28* deletion mutants in *F*. *graminearum*. A, Schematic diagram of the *Fg28* deletion and verification strategy. B, Verification of the candidate *Fg28* knockout mutants by PCR. Mutant strains marked in red were used to evaluate pathogenicity.


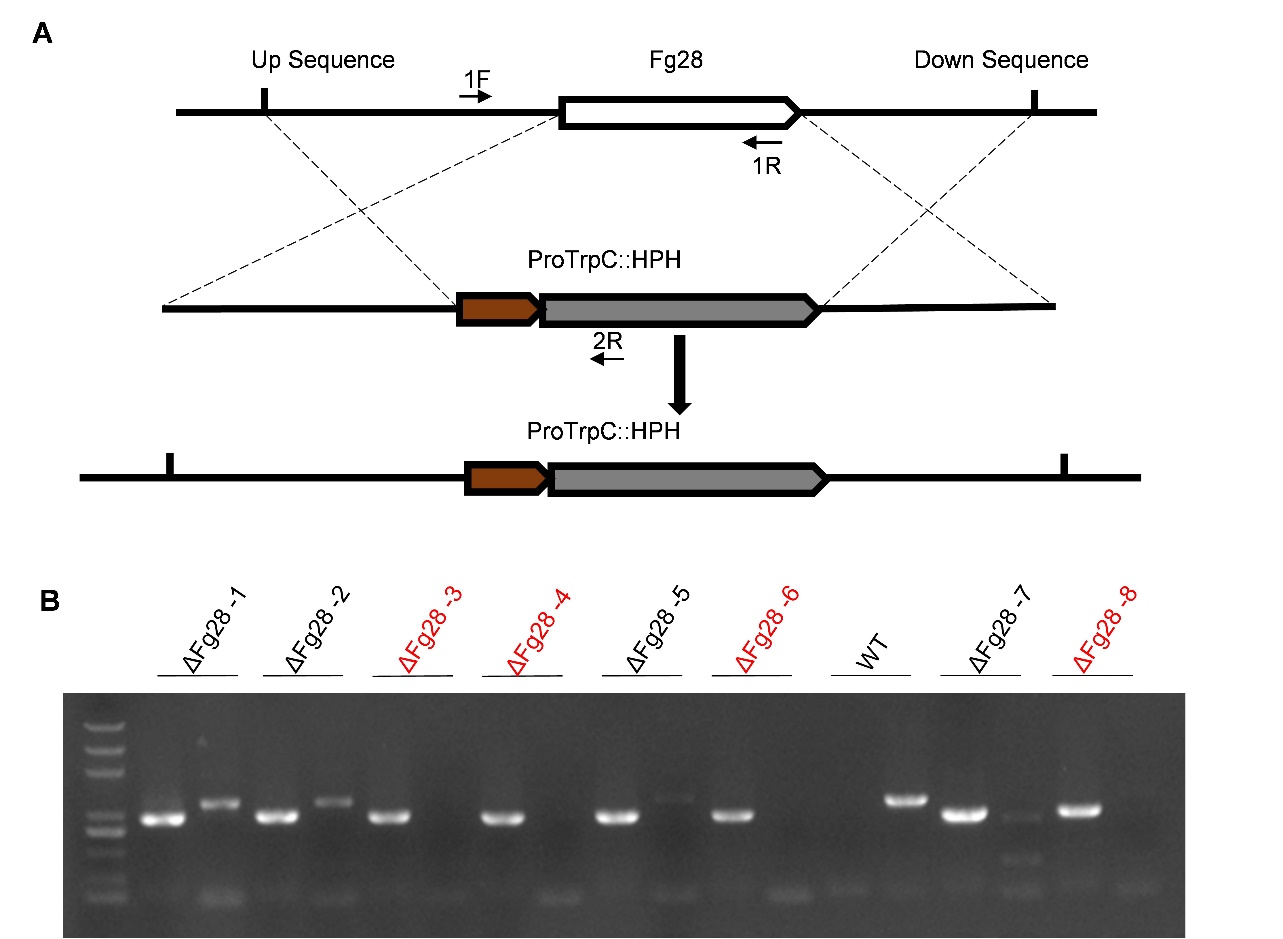

Supplement: Supplementary file 1 [file jof-11-00240-s001.zip › Fg28 Figure S1.docx]
