# Supplementary material for: Proteomic Analysis of the Fusarium graminearum Secretory Proteins in Wheat Apoplast Reveals a Cell-Death-Inducing M43 Peptidase
Source: J Fungi (Basel). 2025 Mar 21;11(4):240. doi: 10.3390/jof11040240 (PMC12027835; doi:10.3390/jof11040240)
Supplement: Supplementary file 1 [file jof-11-00240-s001.zip › Fg28 Figure S2.docx]

**Figure S2.** The expression levels of GFP-Flag protein in *N. benthamiana* leaves were analyzed by Western blot using an anti-Flag antibody, with Rubisco protein detected by Ponceau S staining serving as the loading control. The first lane represents the negative control (tobacco leaves injected with infiltration buffer only), while the second lane corresponds to the positive control (tobacco leaves transiently expressing GFP-Flag).
