# Supplementary material for: Proteomic Analysis of the Fusarium graminearum Secretory Proteins in Wheat Apoplast Reveals a Cell-Death-Inducing M43 Peptidase
Source: J Fungi (Basel). 2025 Mar 21;11(4):240. doi: 10.3390/jof11040240 (PMC12027835; doi:10.3390/jof11040240)

**Figure S3.** Full size images of western blots shown in this study.

Figure 2E


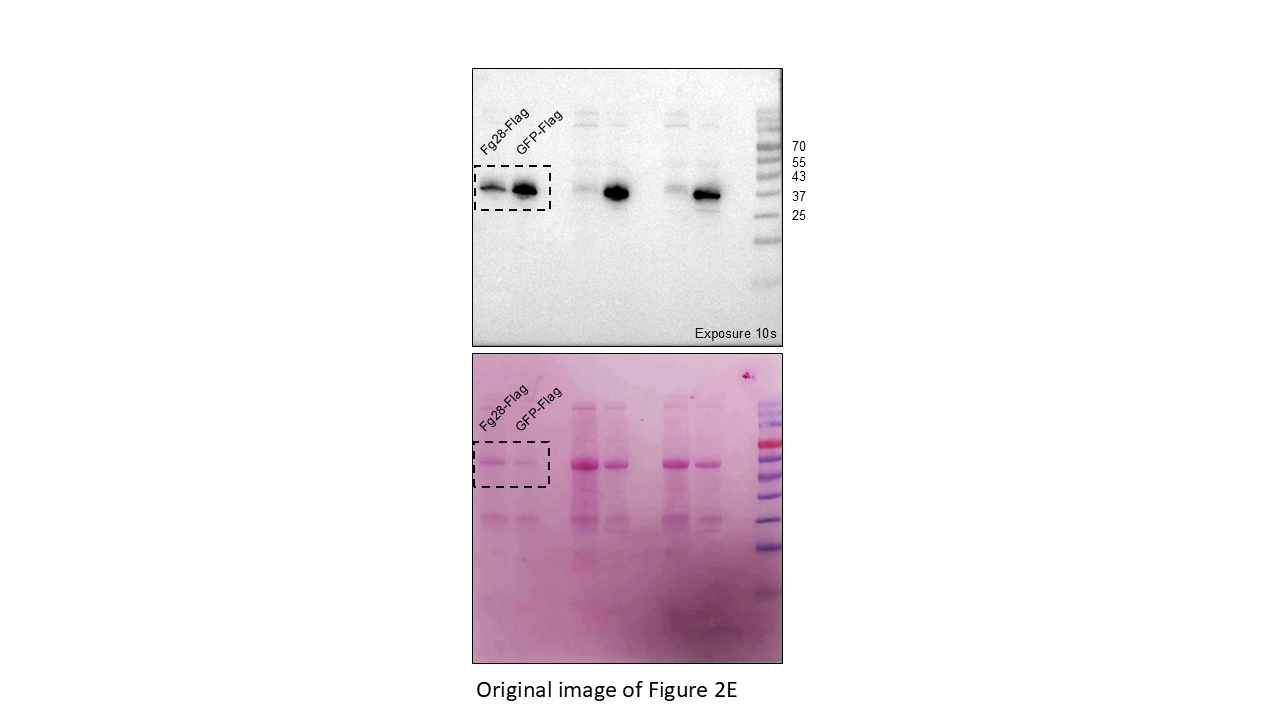


Figure 4D


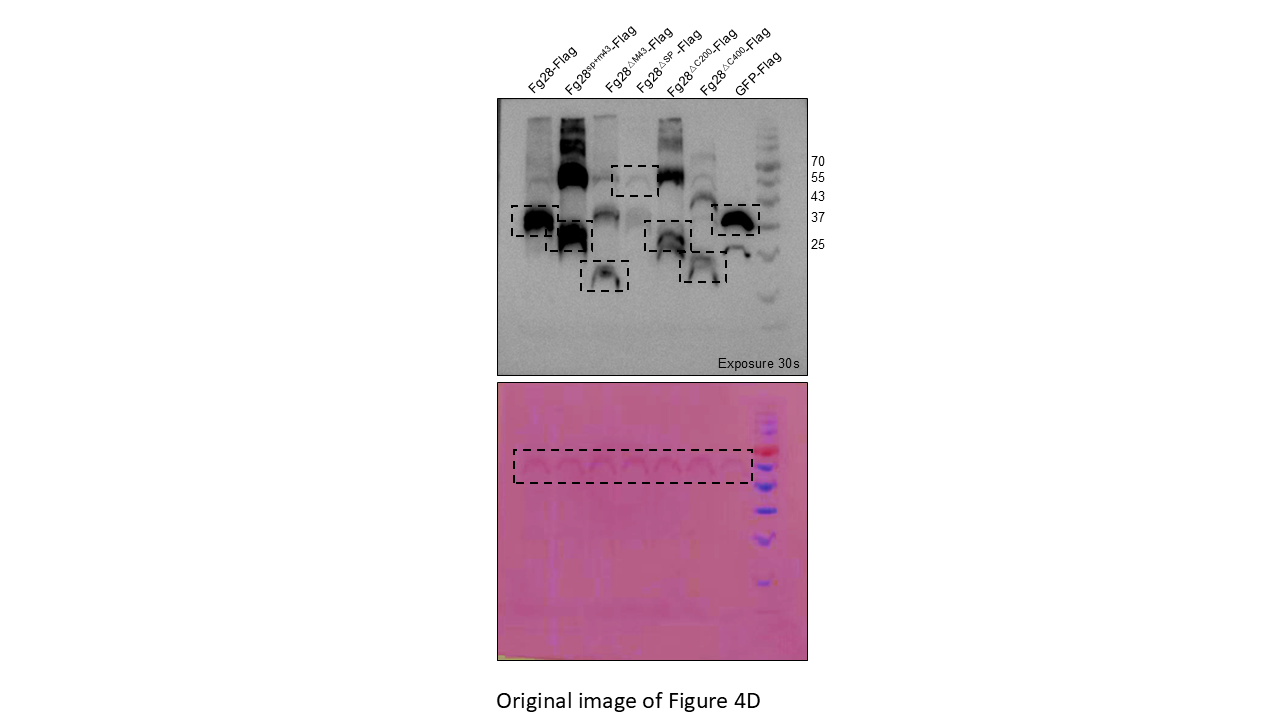


Figure 6B and C


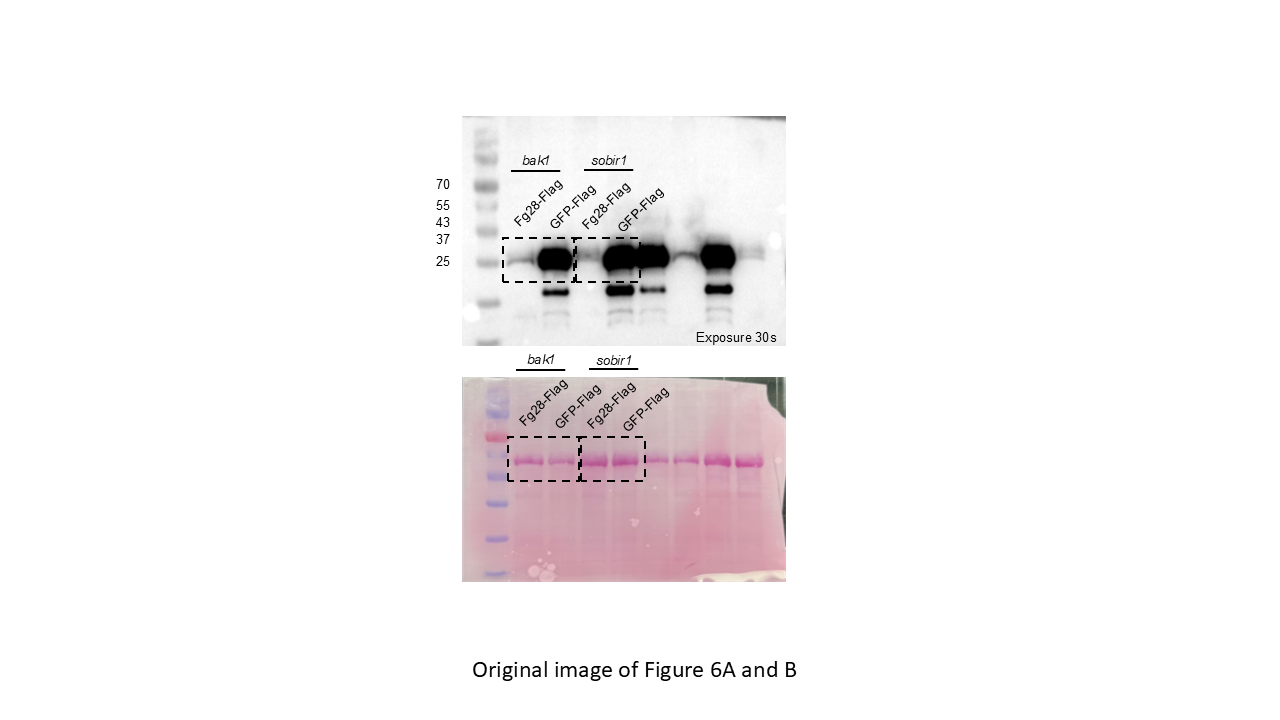


Figure 6D and E


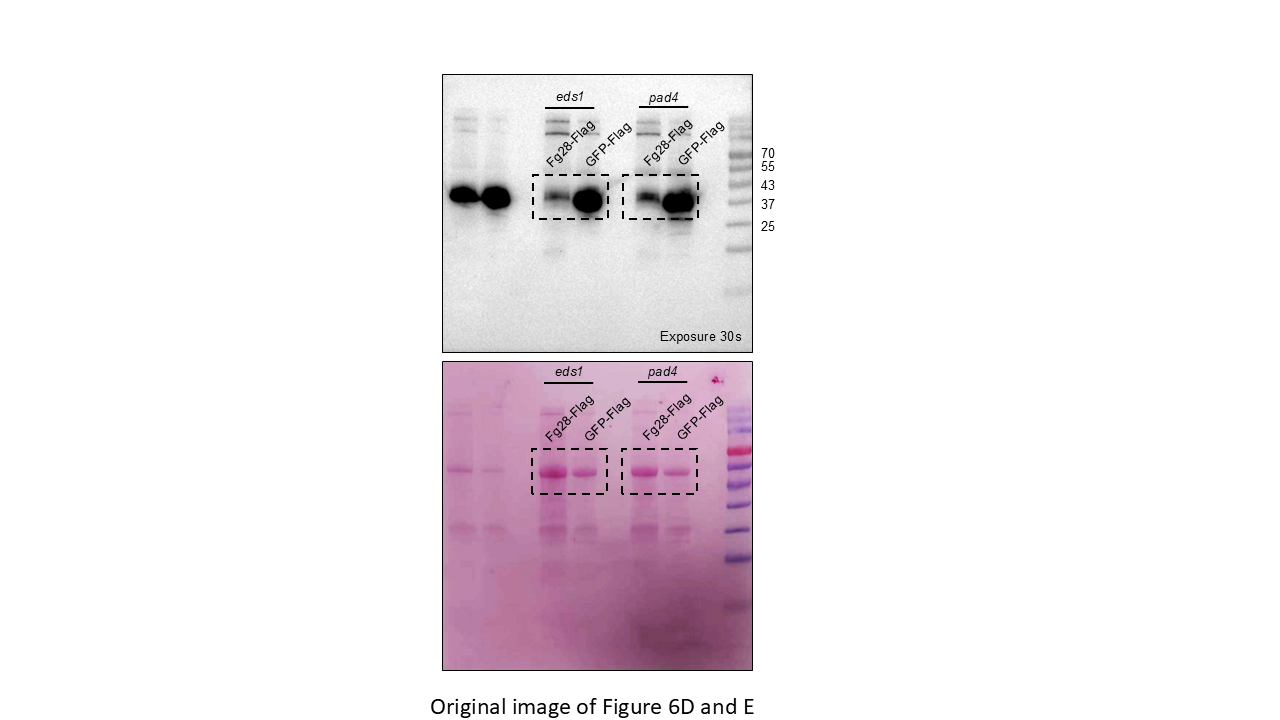

Supplement: Supplementary file 1 [file jof-11-00240-s001.zip › Fg28 Figure S3.docx]
