# Supplementary material for: Proteomic Analysis of the Fusarium graminearum Secretory Proteins in Wheat Apoplast Reveals a Cell-Death-Inducing M43 Peptidase
Source: J Fungi (Basel). 2025 Mar 21;11(4):240. doi: 10.3390/jof11040240 (PMC12027835; doi:10.3390/jof11040240)
Supplement: Supplementary file 1 [file jof-11-00240-s001.zip › Fg28 Figure S4.docx]

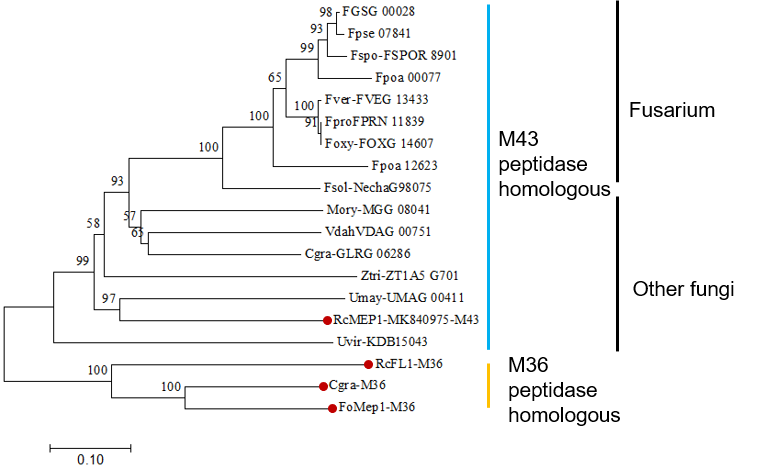


**Figure S4.** Phylogenetic tree of Fg28 and related sequences from selected fungal species. The sequences were obtained from Ensembl Fungi and aligned using MAFFT. The phylogenetic tree was constructed in MEGA with the Neighbor-Joining method. Red circles indicate sequences reported in the published paper.
